# Strengthening health systems response to violence against women: protocol to test approaches to train health workers in India

**DOI:** 10.1186/s40814-020-00609-x

**Published:** 2020-05-11

**Authors:** Sarah R. Meyer, Sangeeta Rege, Prachi Avalaskar, Padma Deosthali, Claudia García-Moreno, Avni Amin

**Affiliations:** 1 Department of Sexual and Reproductive Health and Research, World Health Organization, Geneva, Switzerland; 2 CEHAT–Centre for Inquiry into Health and Allied Themes, Mumbai, India

**Keywords:** Violence against women, Training, Implementation science, Guidelines, Study protocol

## Abstract

**Background:**

Globally, including in low- and middle-income [LMIC] countries, there is increased attention to and investment in interventions to prevent and respond to violence against women; however, most of these approaches are delivered outside of formal or informal health systems. The World Health Organization published clinical and policy guidelines *Responding to intimate partner violence and sexual violence against women* in 2013. Further evidence is needed concerning implementation of the Guidelines, including how health care providers perceive training interventions, if the training approach meets their needs and is of relevance to them and how to ensure sustainability of changes in practice due to training. This manuscript describes a study protocol for a mixed methods study of the implementation of the Guidelines and related tools in tertiary hospitals in two districts in Maharashtra, India.

**Methods:**

The study will employ a mixed-methods study design. A quantitative assessment of health care providers’ and managers’ knowledge, attitudes, and practices will be conducted pre, post, and 6 months after the training. Qualitative methods will include a participatory stakeholders’ meeting to inform the design of the training intervention design, in-depth interviews [IDIs] and focus-group discussions [FGDs] with health care providers and managers 3–6 months after training, and IDIs with women who have disclosed violence to a trained health care provider, approximately 6 months after training. The study will also validate two tools: a readiness assessment of health facilities and a health management information system form in a facility register format which will be used to document cases of violence.

**Discussion:**

The multiple components of this study will generate data to improve our understanding of how implementation of the Guidelines works, what barriers and facilitators to implementation exist in this context, and how current implementation practices result in changes in terms of health services and providers’ practices of responding to women affected by violence. The results will be useful for governmental and non-governmental and United Nations Agency efforts to improve health systems and services for women affected by violence, as well as for researchers working on health systems responses to violence against women in India and possibly other contexts.

## Background

Violence against women is a major public health problem, a gender inequality issue, and a human rights violation. The World Health Organization [WHO] estimates that almost one in three women globally (35%) have experienced physical and/or sexual intimate partner violence [IPV] or non-partner sexual violence [NPSV] [1]. Violence has significant and long-lasting impacts on women’s physical and mental health, including injuries, unintended pregnancy, adverse birth outcomes, abortions (often in unsafe conditions), HIV and sexually transmitted infections, depression, alcohol-use disorders, and other mental health problems [1–5]. Globally, including in low- and middle-income [LMIC] countries, there is increased attention to and investment in interventions to prevent and respond to violence against women, including interventions to address unequal gender norms and acceptability of violence against women that drive violence perpetration [6, 7]. Most of these approaches are delivered outside of formal or informal health systems.

Women who have experienced intimate partner violence and/or sexual violence are more likely to seek health care than non-abused women, even if they often do not disclose the violence to the health care provider [8]. Health care providers, including doctors and nurses, are in a unique position to support women who have experienced violence. However, evidence of effective health-sector responses in LMIC is limited, and there is often little recognition of the role that health care providers and services can play to address violence against women [9]. A majority of evaluations of interventions to improve providers’ response to violence against women are from high-income contexts [10–12], where capacity, systems, and context are substantially different from LMIC health systems. Rigorously evaluated interventions in LMICs include a nurse-led empowerment and counseling intervention in antenatal care in South Africa [13] and a nurse-led intervention in public health clinics in Mexico City [14]. An increasingly widely used model for responding to violence against women has been one-stop centers [15, 16]; however, the effectiveness and sustainability of this model is unknown, and lack of integration within health systems is in some cases a concern.

The World Health Organization published clinical and policy guidelines *Responding to intimate partner violence and sexual violence against women* in 2013 [henceforth, the Guidelines]. The aim was to strengthen health providers’ capacity and improve health system readiness to respond to violence against women [17]. WHO has published two tools to translate the Guidelines into concrete action, with practical “how to” instructions and job aids: a clinical handbook for health care providers, *Health care for women subjected to intimate partner violence or sexual violence* [18] [henceforth, the Clinical Handbook] and a manual for health managers, *Strengthening health systems for women subjected to intimate partner violence or sexual violence* (2017) [19] [henceforth, the Manager’s Manual]. The Guidelines and related implementation tools emphasize the centrality of providing women-centered care. This needs to include, at a minimum: identifying women experiencing violence, providing first-line support/psychological first aid that includes connecting women to other support services they may need, providing comprehensive post-rape care to survivors of sexual assault, and providing basic psychosocial support as part of mental health care. Mental health interventions for those experiencing moderate to severe depression and post-traumatic disorders are also recommended, but may require referral to specialist services.

Training of health care providers is central to efforts to improve the health system response to women affected by violence [20]. A systematic review of training programs to improve health providers’ response to intimate partner violence suggests that training interventions that use interactive techniques are more likely to improve clinical practice of providers [17]. However, there are several other barriers to a quality health system response to violence against women. For example, provider attitudes and bias, whereby health care providers’ own perspectives on violence influence their willingness to ask and how they respond to women affected by IPV [21–23]. Secondly, the literature highlights the perceived lack of self-efficacy and self-confidence among providers to provide adequate care to women survivors, which speaks to the need for further training [24–26]. There are also health system-level constraints, including lack of time to provide adequate support to women during regular clinical practice and inadequate infrastructure [27]. Finally, another barrier is the lack of established referrals and networks with other services, resulting in providers feeling that they are unable to provide adequate to support that women who do disclose or who they identify as subject to violence [27, 28]. Hence, training alone is not sufficient for improving clinical practice; the readiness of the system or services in which providers deliver care also influences their ability to respond [9].

### Study rationale

There are several gaps in evidence that need to be addressed in order to improve understanding of how best to strengthen health system’s response to violence against women and particularly, intimate partner violence. This includes how health care providers perceive training interventions, if the training approach (content and/ or structure of delivery) meets their needs and is of relevance to them, how to ensure sustainability of changes in practice due to training (e.g., using capacity strengthening approaches such as job aids, supervision, and mentoring), and how to strengthen health system readiness (e.g., improvements in infrastructure, referral networks, and documentation systems).

There is also limited understanding of women’s needs and perceptions of quality of care in terms of response to violence against women in the health system in LMIC settings. A meta-analysis of qualitative studies of expectations of women experiencing intimate partner violence found that women wanted the health care provider to display an understanding of the complexity of intimate partner violence, understand its long-term nature (and, hence, the difficulty of a quick resolution), and understand its social and psychological ramifications [29]. The findings, however, all derive from studies conducted in high-income countries; understanding the expectations and needs of women affected by violence from health care providers and services in LMIC settings is therefore critical to develop responses and improve quality of care [30].

The gap in evidence from LMICs is also related to a widespread variation in the instruments used to measure outcomes and impacts of training interventions. Some assessment measures of knowledge, attitudes, and practices of health care providers in relation to responding to violence against women have been developed for high-income settings, such as the Physician Readiness to Manage Intimate Partner Violence Survey [PREMIS] tool validated with physicians in the USA [31]. There are also tools to assess health service/system readiness in the context of response to violence against women [19, 32–34]. These tools contain varying degrees of detail for assessing health system/service readiness and for monitoring and evaluation, and there is a need for a set of validated tools to assess improvements in provider skills and in health system readiness for responding to violence against women in LMIC settings.

Implementation science is “the scientific study of methods to promote the systematic uptake of research findings and other evidence-base practices into routine practice, and, hence, to improve the quality and effectiveness of health services” [35]. There are multiple implementation research frameworks; given the research questions addressed in this study, the approach chosen to guide the study design and selection of research methods is a hybrid effectiveness-implementation study, assessing implementation strategy, and improving understanding of the contextual factors influencing implementation effectiveness [36]. In addition, we will frame the research with systems-thinking within the context of health-systems strengthening, an analytical approach and conceptual framework that has previously been used to assess positive and negative, intended and unintended consequences, on health systems strength, of a complex health system intervention in Zambia [37, 38]. To address some of the gaps identified in how to provide health care to women experiencing violence, the proposed study will apply implementation science methods to identify aspects of implementation of the Guidelines and related tools, in order to improve understanding of local contextual factors influencing intervention outcomes and support future scale-up.

### Study objectives

The objectives of this study are as follows:

1. To explore feasibility of approaches to roll out the training and service delivery improvement activities based on the WHO Clinical Handbook and Manager’s Manual by:
  - assessing needs and priorities of health care providers and managers in responding to violence against women;
  - adapting, implementing the training, and assessing improvements in provider knowledge, attitudes, and practice/skills; and
  - assessing the relevance of the training approaches in meeting the needs of health care providers and identifying barriers and facilitators for health care providers to deliver care to women subjected to violence
2. To understand the perceptions of quality of care of women subjected to violence who have received care from trained health care providers
3. To develop, validate, and refine instruments for measuring health care providers’ performance and health system/service readiness instrument

## Methods

### Study design

**Fig. 1:**
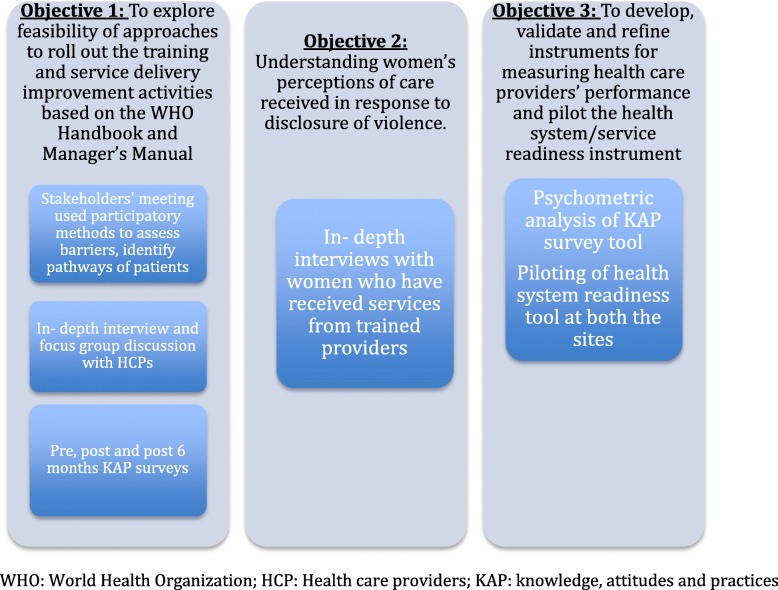
Study objectives and methods

The study will employ a mixed-methods study design, with qualitative and quantitative modes of data collection and analysis to address the objectives. The study design is a single-group feasibility study. A quantitative assessment of health care providers’ and managers’ knowledge, attitudes, and practices will be conducted pre, post, and 6 months after the training. Qualitative methods will include a participatory stakeholders’ meeting to inform the design of the training intervention design, in-depth interviews [IDIs] and focus-group discussions [FGDs] with health care providers and managers 3–6 months after training, and IDIs with women who have disclosed violence to a trained health care provider, approximately 6 months after training. Further description of each method is included below. In addition, the quantitative assessment data from health care providers will be used to validate the instruments for measuring improvements in capacities to deliver care. A readiness assessment of health facilities will also be conducted, and the tool to assess readiness will also be validated and refined based on the data. Another instrument that will be validated will be a health management information system form in a facility register format, which will be used to document cases of violence that are identified or reported and managed by the provider, or referred for management elsewhere. The study processes are summarized and displayed in Fig. 1.

The study has been designed and is being implemented as a partnership between the Centre for Health and Enquiry into Allied Themes [CEHAT], a research non-governmental organization in Mumbai, India; Government Medical College, Miraj; and Government Medical College and Hospital, Aurangabad [Implementing Partners]; and the Human Reproduction Programme [HRP] in the Department of Sexual and Reproductive Health and Research of the World Health Organization [WHO]. The coordination of the research and training of trainers’ activities will be led by CEHAT (principal investigators) and in collaboration with HRP as co-investigators. The implementation of the trainings and health facility readiness improvements will be led by senior health managers and faculty of the two medical colleges—Aurangabad and Miraj.

### Study settings

This study will be conducted in three health facilities, across two districts in the state of Maharashtra, India. Data from the 2015–2016 National Family Health Survey in India indicate that in Maharashtra, 16.4% of urban women and 26.2% of rural women aged between 15 and 49 report ever having experienced spousal violence, while 2.3% of urban women and 3.4% of rural women report ever having experienced violence during pregnancy [39]. This study will be conducted in tertiary level medical teaching hospitals—the Aurangabad Government Medical College and Hospital (GMCH) in the city and district of Aurangabad, the Miraj Government Medical College (GMC) in the city of Miraj in the district of Sangli, and the Sangli District Hospital, which is in the neighboring town of Sangli.^1^

Aurangabad is the fifth largest city in Maharashtra, with a population of over one million. The Aurangabad GMCH is one of the premiere medical colleges in Maharashtra and the biggest tertiary care hospital, administered by the Directorate of Medical Education and Research (DMER). The hospital has 1177 beds, and the average monthly patient flow of approximately 58,000 outpatient visits in 2017.^2^ Miraj, in southern Maharashtra, has a population of over 350,000. The DMER manages both the Miraj Government Medical College, which has 320 beds, and the Sangli district hospital, which has 380 beds.^3^ The average monthly patient flow for outpatient visits in Miraj GMC and Sangli district hospital was approximately 52,000 in 2017.^4^

### Intervention adaptation, development, and implementation

To address Study Objective 1, to “pilot and validate how to roll out the training and service delivery improvement activities,” we first conducted a 2-day stakeholders’ meeting in March 2018, with 30 health care providers with managerial or administrative responsibilities, including doctors, nurses, and social workers, from the three health facilities included in this study. The purpose of the stakeholder’s meeting was to introduce the study, the intervention tools and methods, and generate understanding of the study context (e.g., staffing profile and strength, training needs and gaps, facility infrastructure and procedures, documentation mechanisms). Inclusion criteria to attend the stakeholders’ meeting were that participants were working in one of 3 priority departments selected for this study—obstetrics and gynecology, casualty (i.e., accidents and emergencies), and general medicine. Participants were nominated by the heads of these departments, with guidance to select different types of providers, with sufficient seniority to support and implement changes in clinical practices.

The stakeholders’ meeting employed a range of participatory methods, including patient flow mapping, work flow mapping, and activities to assess barriers and facilitators for health care provider response to violence against women in these specific facilities. This information was used to finalize the plan and timeline to roll out the trainings and other capacity strengthening activities identified as important in this context.

In each site, we will purposively select participants from each cadre—doctors of different levels of seniority (e.g., residents, medical officers, lecturers, and heads of departments) and nurses (including matron, sister in charge). Heads of department from each of the selected departments in the three facilities will select staff for participation in training, with the following as selection criteria: (i) those who most frequently interact with women patients, (ii) those with a past record of interest in activities besides their departmental role, and (iii) those who will not be transferred from their current position for the duration of the study. The initial trainings have been held in both settings (July–November 2018), and follow-up refresher trainings were conducted (February - June 2019).

### Quantitative assessment and analytic plan

Outcomes of the training for health care providers and managers will be assessed using a simple pre- and post-design with a knowledge, attitudes, and practice (KAP) questionnaire, which will be implemented before and after the training and at 6 months post-training. We have developed a KAP instrument, incorporating relevant items from the PREMIS tool and the Domestic Violence Healthcare Provider Survey Scales [DVHPSS], which has been utilized in Uganda and Nigeria [25, 26]. The instrument has been forward and back translated into Marathi, and piloted with a sample of 20 health care providers in a tertiary hospital in Mumbai, to assess comprehensibility, feasibility, and clarity of questions. The results from the pilot test were utilized to reframe some questions, remove some items that did not perform well, and reword items for contextual relevance. For example, the term IPV was removed as it is not utilized in this context and was replaced with domestic violence [DV], and relevant items were added to reflect local legal frameworks, for example, an item on knowledge of the provisions in the 2006 Protection of Women from Domestic Violence Act. The wording of some items was changed to address issues with comprehensibility that arose in the pilot test—for example, the item “It is demeaning to patients to question them about abuse,” was changed to “It is humiliating to patients to question them about abuse” as the word demeaning could not easily be translated or understood by respondents in the pilot test.

The instrument will be self-administered by the trainees immediately prior to and after the training, and by trainees at 6 months after training. The questionnaires will be paper-based, and subsequently entered into Open-Clinica data entry system. Open-Clinica has integrated range checks and logic checks as data entry rules to ensure data quality, integrity, and completeness. Electronically available data are extracted periodically for data management, interim analysis, and for study progress reporting. Each form filled out by the trainees will be checked for quality and completeness by the CEHAT research team, who will be present during the trainings to also document the fidelity to the process and content for the trainings of trainers. Data entry will take place immediately following each phase of data collection, and a sub-sample of 20% of questionnaires will be double entered to ensure quality control.

The following main variables will be assessed for the KAP questionnaire:

i. *Knowledge of violence against women*: Self-reported knowledge of aspects of violence against women, including signs and symptoms, risk factors, and outcomes; for example, one item in this section asks the respondent to indicate whether the following statement is true or false: “Women who experience violence tend to use health services more often than women who do not.”
ii. *Attitudes towards violence against women*: Self-reported attitudes towards violence against women; for example, one item in this section asks respondents to indicate the extent to which they agree with the following statement: “It is acceptable for a husband to beat his wife if she fails to perform her domestic duties.”
iii. *Providers*’ *clinical practice to respond to violence against women*: Providers’ perceptions of preparedness to identify and care for women subjected to violence and self-reported behaviors related to clinical responses to women; for example, one item in this section asks respondents to indicate how prepared they feel to identify a woman who is or has been subjected to domestic violence by signs and symptoms she reports.

The full baseline KAP survey is included as Supplementary File 1.

Exploratory data analysis will be conducted to identify whether baseline levels of attitudes, knowledge, and practice vary by sex, role, and professional background of respondent, and changes between pre- and post-test, and 6-month follow-up will also be explored by relevant socio-demographic variables. We will compare mean levels of knowledge, attitudes, and practices pre, post, and post-6 months using paired *t* tests or Wilcoxon signed rank *t* test (if distribution of paired differences is not normally distributed). We will also compare differences in baseline levels of knowledge, attitudes, and practices by different socio-demographic variables (sex, location, role) using *t* tests. We will document loss to follow-up, defined as participants who complete the pre/post-training survey but not the post-6 month survey. We will analyze if those lost to follow-up are significantly different than those retained in the study on basic socio-demographic variables, to explore potential biases introduced that may impact validity of study findings [40]. Finally, we will use results from the exploratory data analysis to build multivariate models to assess significance of socio-demographic variables (site, sex, age, profession, and department) on changes in mean levels of knowledge, attitudes, and practices of HCPs. Data will be analyzed in SPSS.

In addition, the data will be used to assess various aspects of validity of the instrument, in order to reduce the number of items in the KAP survey and ensure that the scales are validly and reliably measuring relevant constructs. We will assess one aspect of construct validity by assessing convergent validity, assessing correlations of means of scales measuring similar constructs in the survey (i.e., assessing correlation of mean of two different scales for knowledge included in the survey) [41]. We will conduct exploratory factor analysis [EFA] on separate constructs in the survey, to identify (i) sub-scales within the larger constructs, i.e., types of knowledge and specific forms of attitudes, and (ii) identify redundant items or items that do not correlate well with any factor (determined by having low factor-item loadings). We will assess the number of factors to include using Scree plots and Kaiser criterion, and, after extracting the selected number of factors and conducting the appropriate factor rotation to make the factors more identifiable, we will use the following to guide which items to retain: considering factor loadings (with the general rule-of-thumb of including items with a factor loading of greater than .4), inter-item correlations (with the rule-of-thumb of average correlations of .15 to .5), and item-total correlations (with the rule-of-thumb of greater than .5) [42]. We will calculate the Cronbach’s alpha of each sub-scale, to explore the extent to which the sub-scales are measuring a single construct; a threshold of an alpha coefficient of at least .70 is appropriate for the purposes of a validation study [43]. Other aspects of a formal validation study of the scales—for example, criterion validity—are not possible given lack of gold standard measures for knowledge, attitudes, and clinical practice in this context.

### Sampling for the training and quantitative assessment

For purposes of assessing training and validating the quantitative instruments, we estimated a need for 30% of all health care providers, amounting to a total of 170 health care providers across Aurangabad GMCH and Miraj GMC and Sangli district hospitals. Estimating attrition of 20% between the training and post-6 month assessment—either because of drop outs or incomplete forms—the total number of providers who will be trained and from whom data will be collected will be 220 providers. Given this is a feasibility study and is not formally testing hypotheses, the sample size is not based on power calculations; instead, we identified the largest feasible sample to include in the training, based on knowledge and understanding of HCP and health manager’s availability to and interest in attending training on response to violence against women (see Table 1 for sampling).

**Table 1:** Proposed sample sizes for each study objective

		Aurangabad GMCH		Miraj GMC + Sangli Hospital		
	Method	Sample sizes	Total Aurangabad	Sample size	Total Miraj/Sangli	Total number of participants
Objective 1a: stakeholder consultation, group discussion						
Heads of departments, senior doctors, and nurse in charge	Group discussion + participatory mapping	1 (8–10)	1 (8–10)	1	1	20
Objective 1c: assessing training relevance health care providers and health managers						
Doctors	Post-training IDI	6	6	5	5	11
Nurses, social workers, and support staff	Post-training IDI	6	6	6	6	12
	Post-training FGD	1 (8–10)	1 (8–10)	1 (8–10)	1 (8–10)	20
Managers; heads of departments, professors/associate professors	IDI (post-training)	3	3	2	2	5
					Total	28 provider IDI + 4 FGD
Objective 2: understanding women’s perceptions of care received in response to disclosure of violence						
Women identified as being affected by violence	IDI (after provider trainings)	5	5	5	5	10
Objective 1 b and 3: provider training, assessment of changes resulting from training, and validation of the instrument used to measure health care provider performance						
Doctors including managers	Training + pre/post-training, and post-6 month survey	33	30	95	63	
Nurses, social workers, and support staff including managers	Training + pre/post-training, and post-6 month survey	75	32	162	107	
					170 providers	

To ensure and encourage adequate participant attendance at trainings and completion of assessment instruments, we are employing a number of strategies. We sent a letter to the Dean of each medical college requesting that they allow the heads of relevant departments to depute staff from their respective units for training. In addition, trainings were held on hospital/facility premises. Dates were announced in the respective units, and the agenda was pasted on the door of the venue where the trainings were held. For participant retention, selection was requested for those staff that were going to be in the same facility for at least 18 months of the duration of the project. Despite these efforts, several staff who participated in the training were transferred to other facilities, and hence response rate at 6 months refreshers was around 80%. During the analysis, we will compare whether the responses of the 20% who could not attend trainings at post-6 months for the pre and post-training assessments were comparable to that of the 80% of the sample that was able to complete pre-, post-training, and post-6 month assessment.

### Qualitative assessment and analytic plan

Following the implementation of the trainings, we will assess the relevance of the training in meeting the needs of health care providers and managers and identify barriers and facilitators to their ability to provide care to women subjected to violence. Approximately 3–4 months after the training, we will conduct FGDs and IDIs with health care providers and managers who participated in the training to explore: the extent to which the training met their needs, extent to which health care providers are able to practice what they learned, barriers and facilitating factors that affect their ability to put into practice what they learned, any unexpected outcomes of the training intervention, and suggestions for refinement of the activities or for activities to add to the intervention package for the next phase of the study.

Across the sites, we are proposing to conduct a total of 28 IDIs with doctors, nurses, and other support staff and managers. We will conduct 2 FGDs (each approximately 8–10 participants for a total of 16–20 participants) with nursing cadre; FGDs with 10 participants have been found to be feasible and effective in previous research studies in this context. The sample sizes are calculated to ensure adequate representation across the different departments selected for the training, the levels of seniority of health care providers, and different cadres who receive the training. The sample size calculation is based on feasibility, capacity to capture diverse perspectives, and logistics of implementing data collection requiring busy HCPs to participate in further data collection activities. Recruitment for the post-training assessment will be from among the staff who are trained, and selection will reflect diverse cadres of health care providers and managers across the selected sites.

To address Objective 2, “Understanding women’s perceptions of the quality of care received from trained providers,” we will conduct IDIs with a sample of women who disclose violence during a visit with a trained health care provider, approximately 6 months after the training. The interview will focus on understanding women’s experience of care received in relation to their disclosure of violence, whether the providers’ response met their needs, perceptions of what would help or hinder their willingness to disclose violence, and willingness to return for services or refer others to the same provider.

Purposive sampling will be used to select women for IDIs. All women who disclose previous or current experience of violence during a visit with a health care provider who received the training will be asked whether they are willing to participate in an interview, and a sub-sample of women will be selected from this larger sample to participate in an in-depth interview. Women will be selected from patients attending the outpatient departments of the three departments (i.e., obstetrics and gynecology, general medicine, and casualty/emergency) that are to be selected for the training intervention in Aurangabad GMCH and Miraj GMC and Sangli Hospitals. The women will be identified by health care providers, who will ask the woman if she is willing to participate in an interview about her experience receiving health care, and if so, provide the woman’s contact details to the team of data collectors. To protect confidentiality, the woman’s name will not be passed on to the team of data collectors, only a method of contacting the woman. A total of 10 IDIs will be conducted with women who disclose violence to trained health care providers. Women selected will be 18 or older and be able to give informed consent. Women who have a general mental condition that would preclude them from understanding the informed consent process will be excluded from the IDIs.

Validity of qualitative data will be approached by through the lens of trustworthiness, including credibility, which is described as ensuring that the process of data collection is “logical, traceable, and clearly documented” [44]. To ensure credibility, for all qualitative data collection activities, IDIs and FGDs will be audio recorded and transcribed by professional transcribers in the local language (Marathi) and/or English. All FGDs will be facilitated by two data collectors, with one focusing on note taking to ensure tape recordings can be cross-checked against hand written notes of the discussion. Local language transcripts will be translated into English and translations cross-checked for accuracy by professional translator. The research team of the PI (i.e., CEHAT) in India will review all transcripts, and the research team in WHO, HRP, will review select transcripts in English for quality assurance.

Qualitative data analysis will be conducted in two phases. In the first phase, a data analysis workshop will be held in India, with the CEHAT research team and WHO/HRP research team to facilitate a review of the data. This will enable discussions among the research team regarding themes and codes most relevant to the central research questions, moving from a descriptive coding procedure to a more explicitly interpretive and analytical process [45]. The discussion of key themes will be informed by the data collection process and researchers’ knowledge of the local context. Based on findings from the analysis workshop, the research team will develop a draft codebook. Two CEHAT researchers will conduct line-by-line coding on a sub-sample of transcripts, compare coding schemes, and refine the codebook based on this process, to be finalized in consultation with the WHO/HRP research team. Final analysis of the qualitative data will be conducted by the CEHAT research team using Atlas.ti Version 6 [46]—a qualitative data analysis software, based on the finalized codebook.

### Piloting and validation of other monitoring and evaluation instruments

We will also pilot and validate two other monitoring and evaluation instruments that will contribute to future implementation and evaluation of the Guidelines and of the health system response to violence against women in this and other contexts.

#### Health management information system [HMIS] documentation form to record women’s disclosures of violence and monitor provision of care

The facilities selected for this study will introduce, and train providers on, a documentation form adapted from the WHO Health Manager’s Manual to record cases of intimate partner violence and sexual violence and monitor the provision of care to survivors. The form will be made available in the form of a register, and providers will be trained to enter the information in the system immediately. The items included in the register are unique patient identification code, age, marital status, presenting signs and symptoms indicating violence, forms of violence woman facing, and details of formal and informal support services provided by health care provider. Each department will have a designated person who will ensure the confidentiality of the register. The designated individuals are nurses who are responsible for maintaining the registers, and will keep the register in a locked cupboard, with a protocol for handover during change in shifts. A full review of a 1% sample of documentation forms will be done for quality assurance. After 3 months of utilizing the documentation form, the CEHAT research team will compile and analyze quarterly data. Changes in number of cases of violence reported or disclosed over time and the kinds of responses to women disclosing violence, such as first-line support, referrals, and medical care, will be used as proxy measures for quality of care received, based on the assumption that over time, as trained providers become more skilled in asking about violence, and providing appropriate response, more women will disclose violence.

#### Health service readiness assessment [HSR] instrument

In order to pilot this instrument, we will conduct observations of the departments selected or prioritized for this study in each facility and complete the instrument using a short questionnaire to the managers responsible for these departments, record and document review, and direct observation of the facilities. Based on tools piloted in other settings [34] and understanding of structure of local health facilities, we have proposed source(s) of information for each of the items in the assessment instrument—for example, the item “Is there a written protocol/ standard operating procedures [SOP] for provision of health care to women subjected to domestic and/or sexual violence available in the facility?” will be assessed by asking a facility manager and looking at a copy of the SOP if it exists. For piloting the HSR instrument, we are proposing to select the outpatient facilities of the three selected departments in Aurangabad GMCH, Miraj GMC, and Sangli district hospitals for a total of nine data points. Data gathered through observations, record review, and manager interviews will be used to assess the usefulness of the draft instrument, including whether any items need to be removed as they are not relevant in this context or whether items or domains are missing and need to be added. Selected items for the pilot instrument are included as Supplementary File 2.

### Ethics procedures and approvals

Research on violence against women can present a number of ethical and safety concerns. The present study has developed ethics procedures that follow the WHO recommendations in *Ethical and safety recommendations for intervention research on violence against women* [47].

Measures will be taken to ensure confidentiality of all information provided by respondents. All qualitative interviews will be conducted in a private setting within the health facilities. We will find a designated room within the hospital usually used for counseling. If privacy cannot be ensured, the interview will be rescheduled or conducted elsewhere. Interviewers will be given strict instructions on the importance of maintaining confidentiality during their training, and respondents will be informed about the confidentiality procedures as part of the consent process. For both qualitative and quantitative data, all audio tapes, transcripts, translations, and databases will be locked either physically or electronically, with only members of the research team able to access data. All identifying information will be linked to an anonymous number, which will be stored separately from the interviews, focus group discussion transcripts, and completed quantitative training questionnaires.

All respondents will be asked to give their written informed consent as per usual informed consent procedures. (e.g., forms will include information on background of the study, risks and benefits of participation, and confidentiality procedures). If a respondent is illiterate, a literate witness (a person with no connection to the research team) will witness the respondent providing a thumb print to attest that all the information on the informed consent document has been read to the respondent. Members of the research team will be carefully selected and receive specialized training and on-going support, including an opportunity to debrief after interviews. Interviewers will be trained to refer women requesting assistance to available local services and sources of support. Prior to beginning data collection, CEHAT and the medical colleges will develop a clear procedure for referrals for women affected by violence. The referral system will include resources within the hospital system (crisis intervention, medical support, mental health care) and all available relevant external resources. An adverse events protocol has been developed to address issues including if a respondent expresses concern about living in a situation of on-going violence or expresses suicidal thoughts; details of the plan are available upon request.

Study procedures have been reviewed and approved by (i) an independent technical review panel of HRP, Research Project Review Panel [RP2]; (ii) the World Health Organization’s Ethics Review Committee [ERC], which reviews all human subjects research conducted or supported by WHO; (iii) CEHAT’s Program Development Committee as well as an independent review committee of research ethics experts that reviews all research protocols of CEHAT, and (iv) the Department of Medical Education and Research, Maharashtra. There have not yet been significant protocol changes, and any protocol changes must be approved by RP2 and ERC.

All items recommended for inclusion in a study protocol are included in this manuscript and reported in the SPIRIT Checklist (Additional file 3). This protocol version reflects the study design and methodology as of March 2019. Measures to ensure adherence to the training protocol and post-training implementation include that CEHAT team members are present to monitor fidelity at every training and makes regular site visits to the hospital. In addition, members of the HRP team from Geneva have made a total of 4 monitoring visits in the first 12 months of the project, and teleconferences are held regularly (at least once every 6 weeks).

## Discussion

This article describes the study protocol of a formative phase of research focusing on implementation of the WHO Guidelines, Clinical Handbook, and Manager’s Manual to address violence against women, primarily through training of health care providers and managers, in three tertiary facilities in two districts in Maharashtra, India. The multiple components of the research will generate data to improve our understanding of how implementation processes work, what barriers and facilitators to implementation exist in this context, and how current implementation practices, which primarily focus on training of health care providers, result in changes in terms of health services, and providers’ practices of responding to women affected by violence. The results will be useful for governmental and non-governmental and United Nations Agency efforts to improve health systems and services for women affected by violence, as well as for researchers working on health systems responses to violence against women in India and possibly other contexts. The present study will yield important insights that can inform future implementation efforts, as well as form the basis of a future experimental study to assess impact of training on quality of care in this context.

Our review of existing evidence and evaluations indicated that there is a strong rationale for implementation research focusing on efforts to roll-out the Guidelines. A contact with the health system is an important opportunity to identify women affected by violence and offers first-line support and referrals if needed. Given the limited evidence on this from LMIC settings and a paucity of comparable, validated tools to measure progress on health systems’ response to violence against women, this study will fill an important gap. It will provide evidence concerning design, content, and implementation of training, taking into account multiple perspectives of those impacted by the training—managers, providers, and women accessing health services. It will also help identify health system contexts that can enable or inhibit quality care provision.

Limitations to the study design need to be considered when interpreting the potential impact of the findings of the proposed study. Implementation of the Guidelines aims to improve quality of care for women affected by violence; however, we are not at this stage systematically assessing women’s perceptions of quality of care, for example, through an exit survey completed by a randomly selected sample of women attending health services. The small sample size identified for the IDIs among women who disclose will not be able to be generalized to women who utilize health services and disclose violence in other facilities or other contexts, and therefore, our findings on women’s perceptions of quality of care will necessarily be preliminary. However, analysis of the HMIS can give insight into the profile of women identified. This study is a pre-post study design; therefore, changes in knowledge, attitudes, and practices among participants of the HCP training, indicated by changes in KAP survey scores, for cannot be fully attributed to the intervention. Without a control group that does not receive training, it is possible that improvements in clinical practice, for example, could be due to other interventions or other contextual changes influencing HCP’s knowledge, attitudes, and practices, which are unmeasured in this study design [48, 49]. However, this is a formative research phase, and the pre-post study design was deemed to be the most feasible design, allowing insights into validity of instruments, directions of changes for participants in the training, and feasibility and acceptability of aspects of the interventions.

A further limitation is that we have selected tertiary level facilities that have previously worked with CEHAT for this phase of the research, and findings may not be generalizable to secondary and primary level facilities in these and other districts in Maharashtra, or health facilities in other locations. However, choice between primary, secondary, and tertiary health care facilities in these districts is based on proximity, and there is unlikely to be systematic bias, such as wealth level or ethnic background, due to sampling at tertiary level facilities. However, we plan to conduct similar research in other LMIC health facilities in which the Guidelines, and related tools are being implemented, and to adapt training, measurement instruments, and future study design in secondary and primary level facilities, based on findings from this study.

### Dissemination activities

Dissemination of findings will be through workshops with and presentations to management of the health facilities involved in the training. We also plan to hold discussions with the directorate of health services [DHS] who manage district level hospitals and primary health care facilities under the state, so data from the present study can inform future efforts to expand implementation to primary health care settings.

Findings from the study will also be shared in peer-reviewed journals and other local journals in India. Members of the research team will be lead and co-authors on all publications, and there is no intended use of professional writers. Findings will be made available through multiple fora in Maharashtra, nationally in India, and globally by the research team, including through webinars and policy briefs as well as a national dissemination workshop for policy makers and other stakeholders. The present policy environment in India provides an opportune moment for this study as the Government of India has established a National Commission for Safety of Women, which will include a component of health systems response to violence against women in which health care providers from 750 districts will be trained in 2019. Hence, the findings of this study have the potential to contribute to efforts to scale-up health systems response to violence against women in India.

## Supplementary information

**Additional file 1.** Baseline/Pre-training questionnaire for health-care providers

**Additional file 2.** Selected items from HSR assessment tool

**Additional file 3.** SPIRIT 2013 Checklist: Recommended items to address in a clinical trial protocol and related documents

## Data Availability

Data sharing is not applicable to this article as no datasets have yet been generated or analyzed for the current study. The investigators will have access to the final trial dataset, and there are no contractual agreements that limit access to the dataset for investigators. There are no plans for granting public access to participant level data or statistical code.

## Abbreviations

CEHAT: Centre for Health and Enquiry into Allied Themes
DMER: Directorate of Medical Education and Research
DVHPSS: Domestic Violence Healthcare Provider Survey Scales
FGD: Focus group discussion
GMCH: Government Medical College and Hospital
GMC: Government Medical College
HMIS: Health management information system
HRP: Human Reproduction Programme
HSR: Health Service Readiness
IDI: In-depth interview
IPV: Intimate partner violence
KAP: Knowledge, attitudes, and practice
LMIC: Low- and middle-income
NPSV: Non-partner sexual violence
PREMIS: Physician Readiness to Manage Intimate Partner Violence Survey
WHO: World Health Organization

## Footnotes

1 Miraj and Sangli are both in district of Sangli and are considered twin towns as they are contiguous.

2 This is calculated on the basis of 523,107 outpatient visits from January to September 2017.

3 As both Miraj GMC and Sangli district hospital are managed by the same management and have similar departments and staff that move between the two facilities, for purposes of this study, they will be sampled as one site.

4 This is calculated on the basis 466,221 outpatient visits from January to September 2017.
