# Supplementary material for: Strengthening health systems response to violence against women: protocol to test approaches to train health workers in India
Source: Pilot Feasibility Stud. 2020 May 11;6:63. doi: 10.1186/s40814-020-00609-x (PMC7212611; doi:10.1186/s40814-020-00609-x)
Supplement: Supplementary file 1 — Additional file 1. Baseline/Pre-training questionnaire for health-care providers [file 40814_2020_609_MOESM1_ESM.docx]

**Baseline/Pre-training questionnaire** **for health-care providers**

Location: ____________________________

Date: ______ / ­­­_______ / 20______

Day Month Year

Respondent ID number:

**What is this questionnaire?**

- You may indicate your name on this page, however, this is optional. In any case, your answers are confidential and the questionnaires will be safely kept by WHO.
- This questionnaire is about your understanding and perspectives on violence against women. Your responses will be used to help XXX improve this workshop and tools to assist health professionals on providing effective care for women subjected to violence.
- This is NOT a measurement or appraisal of your work performance. Your answers will not affect your work or position in any way. The information collected through this questionnaire will be analysed for the group as a whole, not individually.

We would greatly appreciate your help in responding to these questions. The questionnaire should take approximately 15-20 minutes to complete. If you would like any questions explained or clarified, please do not hesitate to ask your facilitator.

Thank you for your collaboration.

Name (optional):

Name of health facility:

Town/City:

Sex *(please circle)*: 1. Female 2. Male

*[ For WHO/MoH collaborators --* Code: ___________________________ ]

1. What is your job within the facility? *(Check/circle only one option)*

1. Community health worker
2. Medical doctor / Please identify specialty (if any) _________________________________
3. Midwife
4. Nurse
5. Nursing assistant
6. Psychologist or counsellor
7. Social worker
8. Other (please specify): ____________________________________________________________

2. How long have you been practicing clinical work (including your residency)?

_____ Years _____ Months

3. How long have you been working in this facility? _____ Years _____ Months

4. What is the average number of patients (male or female, presenting with any health complaint) you care for per week (*Check/circle only one option*):

1. Currently not seeing patients
2. Less than 20
3. 20-39
4. 40-59
5. 60 or more

5. What is your age group? *(Check/circle only one option)*

1. Less than 25 years old
2. 25-34 years old
3. 35-44 years old
4. 45-54 years old
5. 55 years or older

6a. Have you had any training on violence against women?

a. Yes

b. No –- Please go to question 7

6b. If yes, what kind of training have you had about violence against women? (*Check/circle Yes or No for each option)*

1. Have you watched a video? - Yes/No
2. Have you attended a lecture or talk? - Yes/No
3. Have you attended a skills-based training or workshop ? - Yes/No 🡪 Please go to 6c if YES for any of a, b or c.

6c. If you have participated in a skills-based training, please indicate how long the training way (weeks, days or hours as relevant) and what were the main topics covered.

| Institution that provided training | Year | How long did it last? | Main topics covered |
| --- | --- | --- | --- |
|  |  | Hours __________  Days __________  Weeks ­­­­­­__________ |  |
|  |  | Hours __________  Days __________  Weeks ­­­­­­__________ |  |

7. Please indicate whether you think that the following statements are true or false:

*(For each row, choose one option by checking/circling the corresponding number)*

|  | **True** | **False** | **I don’t know** |  |
| --- | --- | --- | --- | --- |
| 1. Women who experience violence tend to use health services more often than women who do not. | 1 | 2 | 3 |  |
| 1. The majority of rapes of women are committed by strangers. | 1 | 2 | 3 |  |
| 1. There are common injury patterns associated with domestic violence. | 1 | 2 | 3 |  |
| 1. A woman who has been raped always needs immediate health-care. | 1 | 2 | 3 |  |
| 1. Any disclosure regarding domestic violence or sexual violence should be treated confidentially. | 1 | 2 | 3 |  |
| 1. If a health care provider suspects that a woman is or has been subjected to violence, it is helpful to ask her about it. | 1 | 2 | 3 |  |
| 1. If a health-care provider suspects violence but the woman does not acknowledge it, there is nothing he/she could do to help. | 1 | 2 | 3 |  |
| 1. Health-care providers should not pressure patients to disclose that they are living in an abusive relationship. | 1 | 2 | 3 |  |
| 1. In a suspected case of domestic violence, it is advisable for the health care provider talk to both the woman and her partner together. | 1 | 2 | 3 |  |
| 1. The health-care provider should allow a survivor to make her own decisions. | 1 | 2 | 3 |  |
| 1. The health worker must verify how accurate a woman’s story is by asking the alleged abuser or the woman’s friends and family. | 1 | 2 | 3 |  |
| 1. Even when it is not clear what the health-care provider can do to help the woman, one thing he/she can do is to listen to her closely. | 1 | 2 | 3 |  |
| 1. If the woman starts to cry, the health-care provider should immediately end the conversation so she can leave. | 1 | 2 | 3 |  |
| 1. It is important not to share or discuss the woman’s information with anyone unless she consents to it. | 1 | 2 | 3 |  |
| 1. It is a health care provider’s duty to convince the woman subjected to violence to go to the police or the courts. | 1 | 2 | 3 |  |
| 1. Women in abusive relationships may have valid reasons for not leaving. | 1 | 2 | 3 |  |
| 1. Any woman who reports domestic violence must be given information about the Protection Officer | 1 | 2 | 3 |  |
| r. Domestic violence as defined under the Domestic Violence Act is defined as only physical violence | 1 | 2 | 3 |  |
| s. It is important to document the past sexual history of a woman when examining for sexual assault or rape | 1 | 2 | 3 |  |

8. Which of the following are warning signs that a woman may have been subjected to sexual violence or domestic violence? (*Check/circle Yes or No for each option)*

1. Repeated unwanted pregnancy? - Yes/No/ Don’t know
2. Alcohol or drug abuse? - Yes/No/ Don’t know
3. Repeated sexually transmitted infections? - Yes/No/ Don’t know
4. Chronic unexplained pain or conditions (e.g. pelvic, headaches)? - Yes/No/ Don’t know
5. Frequent injuries? - Yes/No/ Don’t know
6. Injuries that do not match the explanation of how they occurred? - Yes/No/ Don’t know
7. Depression, anxiety or chronic stress? - Yes/No/ Don’t know
8. Thoughts, plans or acts of self-harm or (attempted) suicide? - Yes/No/ Don’t know
9. Repeated health consultations with no clear diagnosis - Yes/No/ Don’t know

9. Which of the following are the most appropriate ways to ask about domestic violence? (*Check/circle Yes or No for each option)*

1. “Are you a victim of domestic violence?” - Yes/No/ Don’t know
2. “Has your partner ever hurt or hit you?” - Yes/No/ Don’t know
3. “Does your partner insult you or threaten you?” - Yes/No/ Don’t know
4. “Many women experience serious problems in their relationships. Have you had any difficulties in your relationship?” - Yes/No/ Don’t know
5. “Are you afraid of your boyfriend/husband?” - Yes/No/ Don’t know
6. “Has anyone else in your family ever hurt you, insulted you or threatened you?” - Yes/No/ Don’t know

10. Which of these indicate the most helpful things that a health-care provider may say or do to support a woman subjected to domestic violence or sexual violence? (*Check/circle Yes or No for each option)*

1. “Would you like to tell me more about that?”- Yes/No/ Don’t know
2. “How do you feel about that?” - Yes/No/ Don’t know
3. “Why did you go there alone, don’t you know it’s dangerous?” - Yes/No/ Don’t know
4. “Tell me exactly what he did, you must describe to me all the details.” - Yes/No/ Don’t know
5. “You should not feel so sad, you should feel lucky that you survived”. - Yes/No/ Don’t know
6. “If it’s so bad, you should just leave him”. - Yes/No/ Don’t know
7. “I am worried that the violence may be affecting your health and your children’s health”. - Yes/No/ Don’t know
8. “Trust me, I know that this option will be the best for you”. - Yes/No/ Don’t know
9. “I can help you make a plan for you and your children to be safer in the future”. - Yes/No/ Don’t know
10. “You should go back home and try to keep yourself out of trouble in the future.” - Yes/No/ Don’t know

11. Please indicate the degree to which you agree or disagree with each of the following statements:

*(In each row, choose one option by checking the corresponding number)*

|  | **Strongly disagree** | **Disagree** | **Neither agree nor disagree** | **Agree** | **Strongly agree** |
| --- | --- | --- | --- | --- | --- |
| 1. As a health worker, how I respond to a woman who has suffered violence from a partner or sexual abuse is very important. | 1 | 2 | 3 | 4 | 5 |
| 1. A woman subjected to violence will deny that she has been abused if I ask her about it. | 1 | 2 | 3 | 4 | 5 |
| 1. Domestic violence is a private matter and outsiders should not interfere. | 1 | 2 | 3 | 4 | 5 |
| 1. It is never a woman’s own fault if she is raped. | 1 | 2 | 3 | 4 | 5 |
| 1. If the woman had defended herself, she could have avoided being raped. | 1 | 2 | 3 | 4 | 5 |
| 1. I should not try to convince a woman subjected to domestic violence to leave her violent relationship. | 1 | 2 | 3 | 4 | 5 |
| 1. A woman does not deserve to be abused whether or not she continues to stay with her violent husband/boyfriend | 1 | 2 | 3 | 4 | 5 |
| 1. When interviewing a woman subjected to domestic violence or sexual violence who does not want to talk about details of her story, I should insist. | 1 | 2 | 3 | 4 | 5 |
| 1. I would feel uncomfortable asking a woman about violence | 1 | 2 | 3 | 4 | 5 |

12. Do you think it is acceptable for a man to hit his wife or partner in the following situations?

*(Chose one option in each row by circling the corresponding number)*

|  | **Yes, it is acceptable** | **It is sometimes acceptable** | **No, it is never acceptable** | **I don’t know** |
| --- | --- | --- | --- | --- |
| 1. If she fails to perform her domestic duties. | 1 | 2 | 3 | 4 |
| 1. If she disobeys him. | 1 | 2 | 3 | 4 |
| 1. If she provokes him or makes him very mad. | 1 | 2 | 3 | 4 |
| 1. If she refuses to have sex with him. | 1 | 2 | 3 | 4 |
| 1. If she does not look after the children. | 1 | 2 | 3 | 4 |
| 1. If he suspects that she is being unfaithful. | 1 | 2 | 3 | 4 |
| 1. If he finds out that she is unfaithful. | 1 | 2 | 3 | 4 |

Are there any other situations in which you think it is acceptable for a man to hit his wife or partner? *(Please specify) _________________________________________*

13. Please indicate the degree to which you agree or disagree with each of the following statements

|  | **Strongly disagree** | **Disagree** | **Neither agree nor disagree** | **Agree** | **Strongly agree** |
| --- | --- | --- | --- | --- | --- |
| 1. It is the wife's obligation to have sex with her husband whenever he wants it, except when she is sick or menstruating | 1 | 2 | 3 | 4 | 5 |
| 1. Women and men should share authority in the family | 1 | 2 | 3 | 4 | 5 |
| 1. A woman's most important role is to take care of her home and cook for her family | 1 | 2 | 3 | 4 | 5 |
| 1. It is natural (god intended) that men should be the head of the family | 1 | 2 | 3 | 4 | 5 |
| 1. A wife should obey her husband even if she disagrees | 1 | 2 | 3 | 4 | 5 |
| 1. A woman should be able to spend her own money according to her own will | 1 | 2 | 3 | 4 | 5 |

14. Please indicate the degree to which you agree or disagree with each of the following statements

*(Chose one option in each row by circling the corresponding number)*

|  | **Strongly disagree** | **Disagree** | **Neither agree or disagree** | **Agree** | **Strongly agree** |
| --- | --- | --- | --- | --- | --- |
| 1. Asking patients about domestic violence is an invasion of their privacy. | 1 | 2 | 3 | 4 | 5 |
| 1. It is humiliating to patients to question them about abuse. | 1 | 2 | 3 | 4 | 5 |
| 1. If I ask non-abused patients about domestic violence, they will get very angry. | 1 | 2 | 3 | 4 | 5 |
| 1. I am afraid of offending the patient if I ask about domestic violence. | 1 | 2 | 3 | 4 | 5 |
| 1. Asking about the underlying cause of a patient’s injury does not make a difference to providing quality medical care to the patient. | 1 | 2 | 3 | 4 | 5 |
| 1. The way a couple chooses to resolve a conflict is not my business | 1 | 2 | 3 | 4 | 5 |

15. System support

In providing care to women who experience domestic violence, I have the following resources and support to help me carry out my tasks

1. I have a colleague with whom I can get advice on how to respond to a difficult case of domestic violence if I don’t know what to do – Yes/ No/ I don’t know
2. I can readily look up information (e.g. either a guide or standard operating procedure on how to manage cases of domestic violence) – Yes/ No/ I don’t know
3. I have a private space in the facility where I can talk to the woman confidentially about her abuse. – Yes/ No/ I don’t know
4. My supervisor supports me pro-actively asking my patients or clients about whether they are experiencing domestic violence. – Yes/ No/ I don’t know
5. I have names and contact information of people within this facility to whom I can refer the client who discloses violence for additional counselling or psychosocial support. – Yes/ No/ I don’t know
6. I have names and contact information of people outside the facility to whom I can refer the woman to for addition psychosocial support – Yes/ No/ I don’t know

16. How prepared do you feel for conducting the tasks below in the context of your practice? Select a number from 1 (not at all prepared) to 5 (quite well prepared). *(In each row, choose one option by checking the corresponding number)*

|  | **Not at all prepared** | **Slightly prepared** | **Some-what prepared** | **Sufficiently prepared** | **Quite well prepared** |
| --- | --- | --- | --- | --- | --- |
| 1. Identify a woman who is or has been subjected to domestic violence by signs and symptoms she reports 2. Ask a female patient about whether she has experienced domestic violence | 1 | 2 | 3 | 4 | 5 |
|  | 1 | 2 | 3 | 4 | 5 |
| 1. Provide care to a woman who is or has been subjected to domestic violence | 1 | 2 | 3 | 4 | 5 |
| 1. Offer validating and supportive statements to a woman subjected to domestic or sexual violence | 1 | 2 | 3 | 4 | 5 |
| 1. Talk to the woman about her needs and the options she may have | 1 | 2 | 3 | 4 | 5 |
| 1. Document the history and physical examination findings in patient’s chart | 1 | 2 | 3 | 4 | 5 |
| 1. Assess the immediate level of danger for a woman after sexual assault and/or domestic violence | 1 | 2 | 3 | 4 | 5 |
| 1. Help the woman to create a plan to increase her and her children’s safety | 1 | 2 | 3 | 4 | 5 |
| 1. Refer the woman to support services available within the community (psychological, legal, shelter, etc.) | 1 | 2 | 3 | 4 | 5 |

17. In the past 3 months, have you identified any one suffering from domestic violence among your female patients? (E.g. picked up an acute case, uncovered ongoing abuse, had a woman disclose a past history of domestic violence or sexual violence)

(*Check only one option*)

1. Yes ------- > Approximately how many women? ­________________
2. No ------- > end of questionnaire
3. N/A – not in clinical practice during this period -------> end of questionnaire

18. For the women subjected to domestic violence that you have identified in the past 3 months, which of the actions below have you taken (*Check/circle Yes or No for each option)*

1. Provided basic information about domestic violence to the woman- Yes/No
2. Offered validating and supportive statements- Yes/No
3. Talked to the woman about her needs Yes/ No
4. Discussed the options she may have - Yes/No
5. Documented domestic violence history and physical examination findings in patient’s chart- Yes/No
6. Assessed the immediate level of danger for the woman- Yes/No
7. Helped the woman to create a plan to increase her and her children’s safety- Yes/No
8. Provided education or resource materials about domestic violence to the woman (pamphlets, brochures, etc)- Yes/No
9. Refer the woman to support services available within the community (psychological, legal, shelter, etc.)- Yes/No
