# Supplementary material for: Strengthening health systems response to violence against women: protocol to test approaches to train health workers in India
Source: Pilot Feasibility Stud. 2020 May 11;6:63. doi: 10.1186/s40814-020-00609-x (PMC7212611; doi:10.1186/s40814-020-00609-x)
Supplement: Supplementary file 2 — Additional file 2. Selected items from HSR assessment tool [file 40814_2020_609_MOESM2_ESM.docx]

**Supplementary File 2**

**Selected items from HSR assessment tool:**

| **Items** | **Response Categories** | **Source for information**  **D = direct observation**  **I = Interview**  **R = review of records, reports, protocols.** | **Notes** |
| --- | --- | --- | --- |
| **Section 1: Service delivery/provision** | | | |
| 1. Is there a written protocol/ SOP for provision of health care to women subjected to domestic and/or sexual violence available in the facility? | Neither…………………………. 0  Only sexual violence……… 1  Only domestic violence…. 2  Both……………………………… 3  Don’t Know…………………. 98  Not Applicable…………….. 99 | **I – Facility Manager and unit/department manager**  **D, R** | Note whether available in facility, or department itself or both |
| 1. Where is the written protocol/SOP for provision of health care to women subjected to domestic and/or sexual violence kept? | In the facility administrator’s office….1  In the unit/department office………….2  In the examination room…………….…..3  Other (specify)………………………………...4  Don’t Know………………….98  Not Applicable…………….99 | **I – I - unit/department manager**  **I - Health care provider**  **D** | Randomly ask at least 4 providers in the unit (Junior doctor, senior doctor, nurse) before marking relevant answer  If yes, note where it is stored for access |
| 1. Does the unit/department provide the following aspects of care/management of domestic violence | 1. Ask/identify cases of domestic violence   No………………………………..0  Yes……………………………….1  Don’t Know…………………..98  Not Applicable……………..99 | **I –Facility & unit/dept manager** | You may wish to interview social work unit or if a crisis intervention unit exists about some of these questions. If however, the unit/department managers are not aware of these, please note this. |
|  | 1. Offer psychological support/crisis counselling/first-line support to those who disclose   No………………………………..0  Yes……………………………….1  Internal Referral to other department…..2  Don’t Know…………………..98  Not Applicable……………..99 |  |  |
|  | 1. Inform the survivor of the option of going to a protection office to report domestic violence case   No………………………………..0  Yes……………………………….1  Don’t Know…………………..98  Not Applicable……………..99 |  |  |
|  | 1. Refer to NGOs or any other services outside the health facility that the woman might need.   No………………………………..0  Yes……………………………….1  Don’t Know…………………..98  Not Applicable……………..99  OTHERS- SPECIFY: External referral is done by other internal department |  |  |
| **Section 2: Health workforce** | | | |
| 1. How many staff do you have in this unit/dept? | Doctors  <20………………………………………1  20-50…………………………………….2  >50……………………………………….3  Don’t Know……………………………98 | **I – unit/dept manager& facility manager**  **R** | Ask unit or department or head of facility to provide a written tabulation of staffing per cadre/type of provider. |
|  | Nurses  <50………………………………………1  50-100…………………………………….2  >100……………………………………….3  Don’t Know……………………………98 |  |  |
|  | Counsellors/Social workers  < 5…………………………………………..1  5-10………………………………………..2  >10…………………………………………3  Don’t Know……………………………98 |  |  |
|  | Those with managerial/supervisory responsibilities  Specify number:_________  Don’t Know……………………………98 |  |  |
| 1. How many health-care providers in this unit/dept are designated or given clear responsibility to manage cases of domestic or sexual violence? | Sexual violence:  Doctors:_______  Nurses:_________  Counselors/Social workers:_______  Other (specify)__________________  Don’t know……………………………………98  Not applicable……………………………….99 | **I – unit/dept manager& facility manager** | Ask unit or department manager if provision of care for domestic or sexual violence is included in any written job descriptions or written terms of reference of any staff cadre and if so, ask to review these and note these down. If only indicated orally to relevant staff then also note that. |
|  | Domestic violence  Doctors:_______  Nurses:_________  Counselors/Social workers:_______  Other (specify)__________________  Don’t know……………………………………98  Not applicable……………………………….99 |  |  |
| **Section 3: Infrastructure and medical products:** | | | |
| 1. Is there a space (for example, a room or area) available for private and confidential consultation (that is, that ensures the survivor cannot be seen or heard from outside)? | Neither…………………….…..0  Only visual privacy………..1  Only auditory privacy…….2  Both visual and auditory privacy………………..3  Don’t Know…………………..98 | **I – unit/dept and facility manager**  **D** |  |
| **Does the facility have the essential supplies and equipment? (22 items total) [two sample items provided here]** | |  |  |
| 1. Examination /table couch (with curtains or screens if needed for privacy) | No………………………………..0  Yes……………………………….1  Don’t Know…………………..98  Not Applicable………………99 | **I – unit/dept and facility manager**  **D** | Indicate/note where consultation and examination of the patient are happening if there is no examination table or couch |
| 1. Secure record storage cabinets with a lock | No………………………………..0  Yes……………………………….1  Don’t Know…………………..98  Not Applicable………………99 | **I – unit/dept and facility manager**  **D** | Indicate where and how records are being stored if there is no secure cabinet with locks |
| **Section 4: Leadership, governance and accountability** | | | |
| 1. Are health managers supportive of efforts to address violence against women (e.g. approve staff to spend time caring for survivors, willing to send staff for trainings, willing to make changes in the unit to support care for survivors etc)? | Not at all……………………..0  Somewhat.………………….1  Fully supportive……………2  Don’t Know………………..98  Not Applicable……………99 | **I – unit/dept and facility manager,**  **I - health care providers** | To note this, please randomly select at least 1 senior and 1 junior physician, 1 senior and junior nurse and ask them to rate how supportive is the dept head and other administrators in the facility of providers to care for survivors of violence and also ask them to give concrete examples of what they do to indicate their support for this issue? |
| 1. Is there at least 1 confidential mechanism in the facility to receive feedback from women who use the services, including any complaints or violations of rights in the health facility? | No ……………………………..0  Yes……………………………….1  Don’t Know…………………..98  Not Applicable………………99 | **I – unit/dept and facility manager**  **D, R** | Ask what is in place to get complaints about the services received, rights that are.  Depending on the mechanism, ask to see relevant documents or observe presence of mechanisms |
| **Section 5: Budget and Financing** | | | |
| 1. Is there a budget allocated for care provided to women subjected to violence? (e.g. for training, purchase of supplies for post-rape care, special staff, IEC materials on VAW, job aids, protocols etc) | No ……………………………..0  Yes, < 50,0000 Rs/year……….1  Yes, > 50,000 Rs/year..……….2  Don’t Know…………………..98  Not Applicable………………99 | **I – unit/dept facility manager** | Ask if these funds could be made available from the current hospital operating budget and also ask how much if any was spent the previous year for any – i.e. training staff, purchase of post-rape care supplies, IEC materials on VAW, job aids etc. See if the fund is available but providers are not aware |
| **Section 6: Multi-sectoral coordination and community engagement** | | | |
| 1. Do you have a referral directory with names and contact details of organizations/services that respond to cases of violence? | No………………………………..0  Yes……………………………….1  Don’t Know…………………..98  Not Applicable………………99  OTHERS (specify)___________ | **I – unit/dept and facility manager**  **D** | If not available, where and how referral contact information is kept.  If available, ask when it was last updated (i.e. within the last year or earlier). Also note where it is kept and ask whether staff are aware it exists |
| 1. Is there a referral system across the different departments/units (e.g. ob/gyn and forensics or casualty) within the health facility to receive and manage survivors of sexual or domestic violence? | No ……………………………..0  Yes……………………………….1  Don’t Know…………………..98  Not Applicable………………99 | **I –unit/dept and facility manager**  **R** | Note (review)if there are:  Referral forms  Agreement to prioritize violence survivors  Efforts to phone providers in other units about the referral  Any other approaches |
| **Section 7: Information, monitoring and evaluation** | | | |
| 1. How does the facility/unit document cases or incidents of domestic violence or sexual violence? | There is no documentation system…..0  In a facility register (separate for VAW or as part of another health register…………1  In electronic medical records as part of patient records……………………………………………2  Other (specify)………………………………..3  Specify_________________________  Don’t know…………………………………….98  Not applicable………………………………..99 | **I – unit/dept and facility manager**  **R** | Review registers or other types of documentation |
| 1. How does the facility/unit maintain confidentiality of the documentation of cases of violence? | The facility does not have any method for maintaining confidentiality of documentation of violence……………………………………………..0  Identifying information is removed or kept separate from incident record…………….…1  Records, registers and forms are kept in a secure storage with lock and pre-determined access……………………………………………………2  Electronic medical records are password protected with pre-determined access for those who need to care for survivors……3  Any take home cards or information does not have any direct indication of the survivor’s abuse…………………………………………………….4  All of 1, 2, 3 and 4…………………………………5  Other……………………………………………………6  Specify____________________________  Don’t know…………………………………………..98  Not applicable………………………………………99 | **I – medical officer/ facility manager**  **R** | Circle all the methods that are relevant. If all items 1 to 4 are in place then circle 5.  In addition to interviewing the facility or dept manager, ask to see records, registers and electronic medical records interface if relevant |

SOP: Standard Operating Procedures
